# Supplementary material for: Correction: The efficacy of machine learning models in lung cancer risk prediction with explainability
Source: PLoS One. 2024 Sep 12;19(9):e0310604. doi: 10.1371/journal.pone.0310604 (PMC11392339; doi:10.1371/journal.pone.0310604)
Supplement: S2 File — (PDF) [file pone.0310604.s002.pdf]

PLOS ONE

RESEARCH ARTICLE

The efficacy of machine learning models in lung cancer risk prediction with explainability

Refat Khan Pathan[1], Israt Jahan Shorna[2], Md. Sayem Hossain[3], Mayeen Uddin Khandaker[4,5], Huda I. Almohammed[6]*, Zuhal Y. Hamd[6]

1 Department of Computing and Information Systems, School of Engineering and Technology, Sunway University, Selangor, Malaysia, **2** Shamsun Nahar Khan Nursing College, Chattogram, Bangladesh, **3** School of Computing Science, Faculty of Innovation and Technology, Taylor's University Lakeside Campus, Selangor, Malaysia, **4** Applied Physics and Radiation Technologies Group, CCDCU, School of Engineering and Technology, Sunway University, Selangor, Malaysia, **5** Faculty of Graduate Studies, Daffodil International University, Daffodil Smart City, Savar, Dhaka, Bangladesh, **6** Department of Radiological Sciences, College of Health and Rehabilitation Sciences, Princess Nourah bint Abdulrahman University, Riyadh, Saudi Arabia

* Hialmohammad@pnu.edu.sa

Check for updates

OPEN ACCESS

Citation: Pathan RK, Shorna IJ, Hossain M.S, Khandaker MU, Almohammed HI, Hamd ZY (2024) The efficacy of machine learning models in lung cancer risk prediction with explainability. PLoS ONE 19(6): e0305035. https://doi.org/10.1371/journal.pone.0305035

Editor: Mohammad Amin Fraiwan, Jordan University of Science and Technology Faculty of Computer and Information Technology, JORDAN

Received: April 16, 2024

Accepted: May 22, 2024

Published: June 13, 2024

Copyright: © 2024 Pathan et al. This is an open access article distributed under the terms of the Creative Commons Attribution License, which permits unrestricted use, distribution, and reproduction in any medium, provided the original author and source are credited.

Data Availability Statement: The dataset used in this work is publicly available at https://data.world/cancerdatahp/lung-cancer-data. The code is publicly available at https://github.com/refatkhan9/lung_cancer_xai/.

Funding: This study was supported by Princess Nourah bint Abdulrahman University Researchers Supporting Project number (PNURSP2024R49), Princess Nourah bint Abdulrahman University, Riyadh, Saudi Arabia in the form of a grant

Abstract

Among many types of cancers, to date, lung cancer remains one of the deadliest cancers around the world. Many researchers, scientists, doctors, and people from other fields continuously contribute to this subject regarding early prediction and diagnosis. One of the significant problems in prediction is the black-box nature of machine learning models. Though the detection rate is comparatively satisfactory, people have yet to learn how a model came to that decision, causing trust issues among patients and healthcare workers. This work uses multiple machine learning models on a numerical dataset of lung cancer-relevant parameters and compares performance and accuracy. After comparison, each model has been explained using different methods. The main contribution of this research is to give logical explanations of why the model reached a particular decision to achieve trust. This research has also been compared with a previous study that worked with a similar dataset and took expert opinions regarding their proposed model. We also showed that our research achieved better results than their proposed model and specialist opinion using hyperparameter tuning, having an improved accuracy of almost 100% in all four models.

Introduction

Lung cancer is one of the most commonly diagnosed cancers worldwide to date. It's a severe disease that affects individuals from all aspects of life regardless of their lifestyle and surroundings environment. This cancer lies within the intricate network of the human respiratory system and arises as a silent menace, often remains unnoticed until it reaches an advanced stage [1]. It can manifest in various forms, from the insidious creep of small cell lung cancer to more common non-small cell lung cancer, presenting its own challenges in diagnosing and treatment. The impact is profound, not just physically but emotionally and socially as well. One's journey through lung cancer is often fearful and uncertain, having to make some tough

awarded to H.I.A. The specific roles of this author are articulated in the 'author contributions' section.

Competing interests: The authors have declared that no competing interests exist.

decisions. Starting from a strategic decision about the risk stratification of the screening program to decide eligibility [2, 3].

In the United States, lung cancer is the second most common cancer in both men and women. The American Cancer Society estimates that there will be 234,580 new cases of lung cancer, including 116,310 in men and 118,270 in women, and about 125,070 deaths due to this in the year 2024 [4]. They also reported that the proportion of developing lung cancer in men is 1:16, whereas in women, it is 1:17, including both smokers and non-smokers, but for smokers' people, the chances are much higher. Having a five-year relative duration to gather survival rate, the Surveillance, Epidemiology, and End Results (SEER) database shows 28% relative survival probabilities for non-small cell lung cancer and 7% survival probabilities for small cell lung cancer [5]. Data collected by Cancer Research UK (2016–2018) shows that lung cancer is the third most common cancer in the UK, with around 48500 new cases in the UK every year, including 23,300 cases in women and 25,300 cases in men and only 10% survival rate is remoted in 2013–2017 [6]. In Asian countries like China, a total of 19.2% of cancer-related deaths were reported due to lung cancer in 2017 [7]; India had 8.1% of cancer-related deaths [8]; Malaysia reports that lung cancer has a 10% contribution to all malignancies [7]. Analysing these situations, the seriousness of the prevention and early diagnosis can easily be understood.

Lung cancer has many risk factors; smoking is number one, as reported by the Centers for Disease Control and Prevention (CDC) [8]. According to the CDC, smoking is linked to 80–90% of lung cancer deaths in the USA. Secondhand smoking, which means people who don't smoke themselves but are exposed to smoking, are also highly prone to lung cancer. As reported by the CDC, around 14 million children were affected from 2013 to 2014. The second leading cause of lung cancer is Radon, which is a gas that forms in rocks, soil, and water and can enter buildings through cracks and cause lung cancer if breathed for a long time. Other factors include radiation therapy, abnormal diet, and family genetic history. Risk prediction is more important in lung cancer screening than clinical assessment, as demonstrated by many trials, including the Back model [9], the Lung Cancer Risk Assessment Tool (LCRAT) [10], the Lung Cancer Death Risk Assessment Tool (LCDRAT) [10], the Liverpool Lung Project (LLP) model [11, 12], and the PLCOm2012 model [13, 14].

This study addresses the relationship between lung cancer factors and early symptoms. Four machine learning models are tuned to detect the low, medium, or high lung cancer risk level. Most of the studies only do detection and provide no explanation due to the black-box nature of machine learning. This study overcame that limitation by explaining each model's interpretability using different explanation methods such as decision boundaries, Local Interpretable Model-agnostic Explanations (LIME), and tree extraction. The primary motivation of this study is to explain model results to non-technical people or patients so they can trust the process more. Significant contributions are mentioned in below points:

1. Exploring dataset to figure out relations between different features.
2. Tuning four machine learning algorithms to outperform previous best results.
3. Explaining the model behaviour and reasoning through explainable AI methods.

The rest of the paper is divided into multiple sections, including a literature review in section 3, materials and methods in section 4, result analysis in section 5, discussion in section 6, limitations in section 7 and conclusion in section 8.

Literature review

Though the development in medical science has advanced so far, cancer remains a highly critical and significant concern throughout medical aid, oncology, health care professionals, and AI-based medical science researchers. Diagnosis of lung cancer mainly relies on manual pathology screening, which is highly prone to error due to the human nature of manual film reading. A good number of algorithms and methods were developed using machine learning (ML) and deep learning (DL) to identify cancer from numerical datasets or image-based datasets [15]. Early detection is still crucial for improving survival rates among patients.

Various ML and DL-based techniques have been applied to identify many kinds of cancer diagnosis, prognosis, and risk factors [16–19]. Specifically, AI has been seen to be applied to lung cancer risk assessment, utilising diverse data sources such as medical imaging, genetic markers, clinical records, and environmental factors [20]. ML models incorporating clinical data, such as patient demographics, smoking history, and symptoms, have demonstrated efficacy in predicting lung cancer risk [21]. Among different models, the Support Vector Machine (SVM) showed the highest detection accuracy, but no reasoning has been provided, lacking model explainability. Four ML models, SVM, Naïve Bayes (NB), Decision Tree (DT), and Logistic Regression (LR), were applied to predict lung cancer from two datasets (collected from UCI and Data World), achieving the highest accuracy of 96.9% with LR on UCI and 99.2% with SVM on Data World [22]. In both cases, no model explainability has been provided. Using five different data mining techniques: SVM, K-Nearest Neighbors (KNN), NB, DT, and Artificial Neural Network (ANN), lung cancer prediction has been done with three case scenarios [23]. The best accuracy achieved was 93% using the ANN algorithm and SMOTE Upsampling technique on an unbalanced Kaggle dataset [24]. Biomarkers are also used to identify early lung cancer by analysing the combination of metabolism factors with ML methods. Among the used models, Neural Network (NN) and NB achieved 100% classification accuracy [25]. Among these machine learning models, none was found that explained the internal reasoning that resulted in the accuracy, causing the trustfulness of the application. A customised Lung Cancer Prediction Tool (LCPT) has been developed to predict lung cancer using the risk factor and compared with expert opinion to verify the result [26]. They have shown an accuracy of 93.33% using LCPT, more significant than the specialist opinion of 86.66%. A Random Forest (RF) model was also used to generate ten random trees to compare the results with LCPT. They explained how the factors resulted in the decision using Degree of Importance (DOI).

Image processing of computed tomography (CT) screening has widely been used to diagnose lung cancer using different computer vision techniques [27]. Previously, 2D images of CT were hugely popular for classification and segmentation. As computational power has increased, people are now exploring 3D images and have achieved excellent results. Researchers are using DL methods to identify high-risk smokers suitable for lung cancer screening CT using chest radiographs. Their model's performance was validated, showing promise in improving the selection process of lung cancer screening [28]. A customised deep CNN has been proposed to classify interstitial lung diseases (ILDs) from CT image patches, achieving around 85.61% accuracy, higher than VGG-Net performance, which gained 78% [29]. 3D-VNet and 3D-ResNet architecture have been developed to train 3D CT image slices for segmentation and classification problems [30]. They have achieved a 99.3% Dice Similarity Coefficient (DSC) for segmentation and 99.2% accuracy for classification on the LUNA16 dataset. Though their accuracy and segmentation results are promising, model explainability is very difficult to show due to the complexity of CNN architecture. Another study developed a cascade 3D-UNet to detect lung cancer bone metastases (LCBM) from CT images. Compared

with five radiologists, their model outperformed in detecting LCBM, with higher AUROC (0.875 vs. 0.819) and sensitivity (0.894 vs. 0.892) in an observer-independent study [31]. Combining with transformer and U-Net, an architecture has been developed named UNETR and used to segment 3D images of lung cancer using the Decathlon dataset [32], achieving an accuracy of 97.83% with DSC of 96.42% and a classification result of 98.77% [32]. They have shown the different performances regarding different hyperparameters like optimiser, number of epochs, and activation functions. Still, the CNN explainability remains unnoticed. An ensemble multi-view 3D CNN model has been designed for risk stratification of invasive lung adenocarcinoma using thin slice CT scan images, with an AUC of 91.3% for benign/malignant diagnosis and 92.9% for pre-invasive/invasive nodule classification [33]. It also outperforms senior doctors in risk assessment, having 77.6% accuracy, but lacks information on why it can outperform doctors.

Explainable AI, or XAI, is gaining significant attention because of its ability to understand the reasoning behind the model's prediction, classification, or segmentation. Various works are seen to explain the AI model to understand the importance of features; for example, in chronic wound images, LIME has been applied to understand ROI [34]. Another custom XAI diagnostic model has been proposed to interpret the model using TabNet with causal graphs on mammography reports of breast cancer [35]. Another ensemble learning framework with XAI has been developed by ref. [36] to determine breast cancer with explanations. The SHAP model has been used to explain lung cancer reasoning from biomarker values identified from CT scan reports by ref. [37], where they developed an AI CAD model using multiple ML methods. Many other works have used the XAI method to explain model behaviour on lung cancer detection on both ML and DL methods [38–41].

Regarding the risk factors, almost all image-based detection is the diagnosis of lung cancer, and most risk factors are considered in a numerical form of data with different vital factors and symptoms. This research is based on exploring the relationship between these factors and lung cancer chances using four popular machine learning models named SVM, KNN, DT, and RF. It also explains why the model behaved as it did. This work will improve a previous work [26] that addresses issues on the same dataset and proposes a custom LCPT model. In this work, we have also shown that higher classification accuracy can be achieved using parameter tuning.

Materials and methods

Dataset description

The dataset is taken from Data World [42]. There are 22 features: Age, Gender, Air Pollution, Alcohol use, Dust Allergy, Occupational Hazards, Genetic Risk, Chronic Lung Disease, Balanced Diet, Obesity, Smoking, Passive Smoker, Chest Pain, Coughing of Blood, Fatigue, Weight Loss, Shortness of Breath, Wheezing, Swallowing Difficulty, Clubbing of Fingernails, Frequent Cold, Dry Cough, Snoring. The details of the dataset have been shown in Table 1. The association of features with risk-level classes is significant. It has been seen that people aged around 35–40 are more likely to have lung cancer. Also, high air pollution levels cause a higher risk, along with alcohol use. People with less alcohol use are seen to have lower chances of getting lung cancer. A similar trend has been seen among other features; having high points generally leads to higher positive risk, except shortness of breath, wheezing, clubbing fingernails, and snoring. The exceptions are seen to have a range of values having higher risks. A total feature distribution histogram has been shown in Fig 1.

The dendrogram represents a hierarchical clustering of various features based on their similarity or dissimilarity. The y-axis measures the distance between clusters, with lower values

Table 1. Dataset description.

Feature	Count	Mean	Standard deviation	Min	25%	50%	75%	Max
Age	1000	37.17	12.00	14	27.75	36	45	73
Gender	1000	1.40	0.49	1	1	1	2	2
Air Pollution	1000	3.84	2.03	1	2	3	6	8
Alcohol use	1000	4.56	2.62	1	2	5	7	8
Dust Allergy	1000	5.16	1.98	1	4	6	7	8
Occupational Hazards	1000	4.84	2.10	1	3	5	7	8
Genetic Risk	1000	4.58	2.12	1	2	5	7	7
Chronic Lung Disease	1000	4.38	1.84	1	3	4	6	7
Balanced Diet	1000	4.49	2.13	1	2	4	7	7
Obesity	1000	4.46	2.12	1	3	4	7	7
Smoking	1000	3.94	2.49	1	2	3	7	8
Passive Smoker	1000	4.19	2.31	1	2	4	7	8
Chest Pain	1000	4.43	2.28	1	2	4	7	9
Coughing of Blood	1000	4.85	2.42	1	3	4	7	9
Fatigue	1000	3.85	2.24	1	2	3	5	9
Weight Loss	1000	3.85	2.20	1	2	3	6	8
Shortness of Breath	1000	4.24	2.28	1	2	4	6	9
Wheezing	1000	3.77	2.04	1	2	4	5	8
Swallowing Difficulty	1000	3.74	2.27	1	2	4	5	8
Clubbing of Fingernails	1000	3.92	2.38	1	2	4	5	9
Frequent Cold	1000	3.53	1.83	1	2	3	5	7
Dry Cough	1000	3.85	2.03	1	2	4	6	7
Snoring	1000	2.92	1.47	1	2	3	4	7

https://doi.org/10.1371/journal.pone.0305035.t001

indicating greater similarity and likeliness of occurring together. Each feature on the x-axis corresponds to a symptom or factor of lung cancer. Feature distance has been calculated using a dendrogram graph and is shown in Fig 2. As we move up the hierarchy, clusters form by joining related symptoms and factors. For example, Fatigue and Snoring suggest that they are likely to occur together. Broader clusters emerge as we ascend the dendrogram, where Age and Gender show that they are unlikely to be related to each other. The red cluster indicates a close relation with risk level and other highly connected features.

Another correlation matrix has been generated to show the relationship between features in a better way in Fig 3. Light color represents a higher correlation, and dark color means a lower correlation. It can be noticed that Age and Gender have the lowest correlation with Levels of 0.079 and -0.16, and Obesity and Coughing of blood have higher correlations of 0.82 and 0.77. Among features, a high (0.82–0.88) correlation value is seen among Occupational hazards, Genetic risk, Alcohol use, and Dust Allergy. Some more high correlations between 0.79–0.82 are seen among Chest pain and Occupational hazard, genetic risk, lung disease, and balanced diet. The lowest correlation value is seen among smoking and weight loss as -0.27.

Model training and validation

Four widely known ML models (SVM, KNN, DT, RF) are selected to train and validate the data to predict the risk factors. The dataset has been split into train and test, with 70% for training and 30% for testing, which included 219 low, 235 medium, and 246 high-risk level datapoint in training and 84 low, 97 medium, and 119 high-risk level datapoint in test portions (details shown in Table 2). The model training speed is fast, and it took around 1–2 seconds to

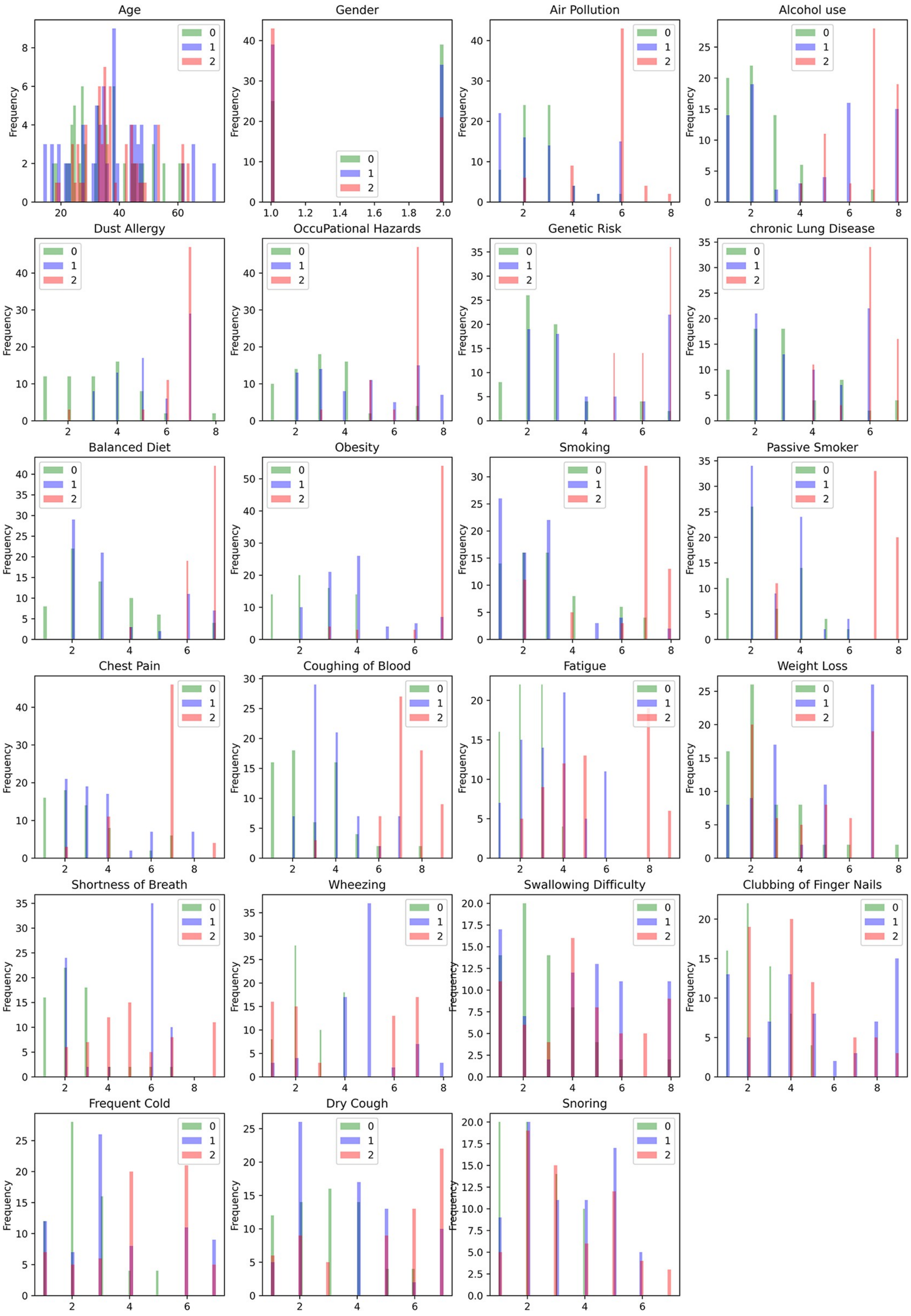

Fig 1. Feature distribution histogram, where 0 = low, 1 = medium and 2 = high.

https://doi.org/10.1371/journal.pone.0305035.g001

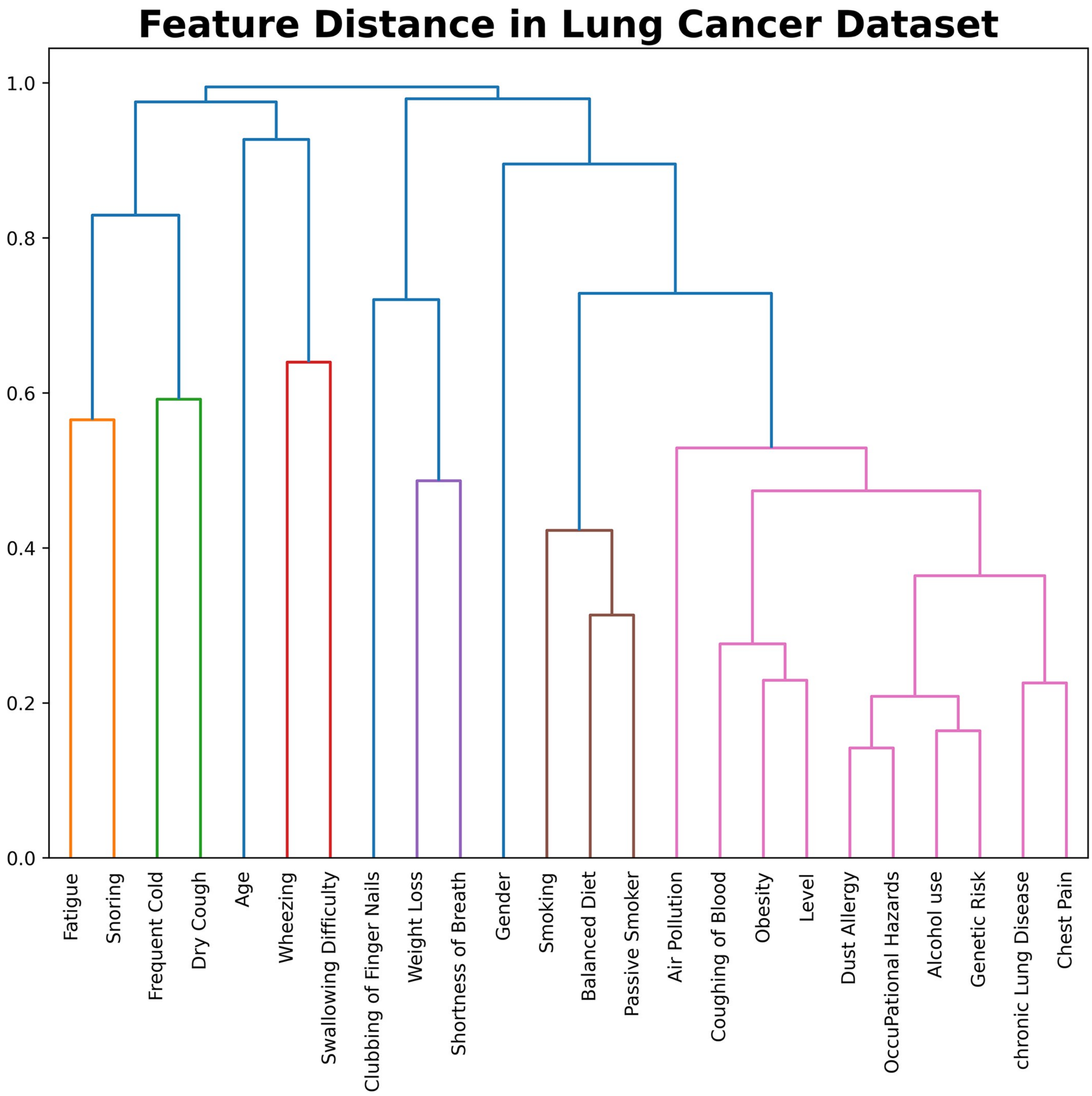

Fig 2. Feature distance calculation using a dendrogram graph.

https://doi.org/10.1371/journal.pone.0305035.g002

train all four models. From the previous dataset analysis section, looking into the correlation matrix, it can be understood that Age and Gender might not have a major contribution to the risk factor. The Random Forest classifier is used to calculate the feature importance, having n_estimator as 20. It also shows that Gender has 0 feature importance and Age is the second lowest with a 0.0022 score. Hence, these two features have been deducted from the training dataset. The total feature importance is shown in Table 3.

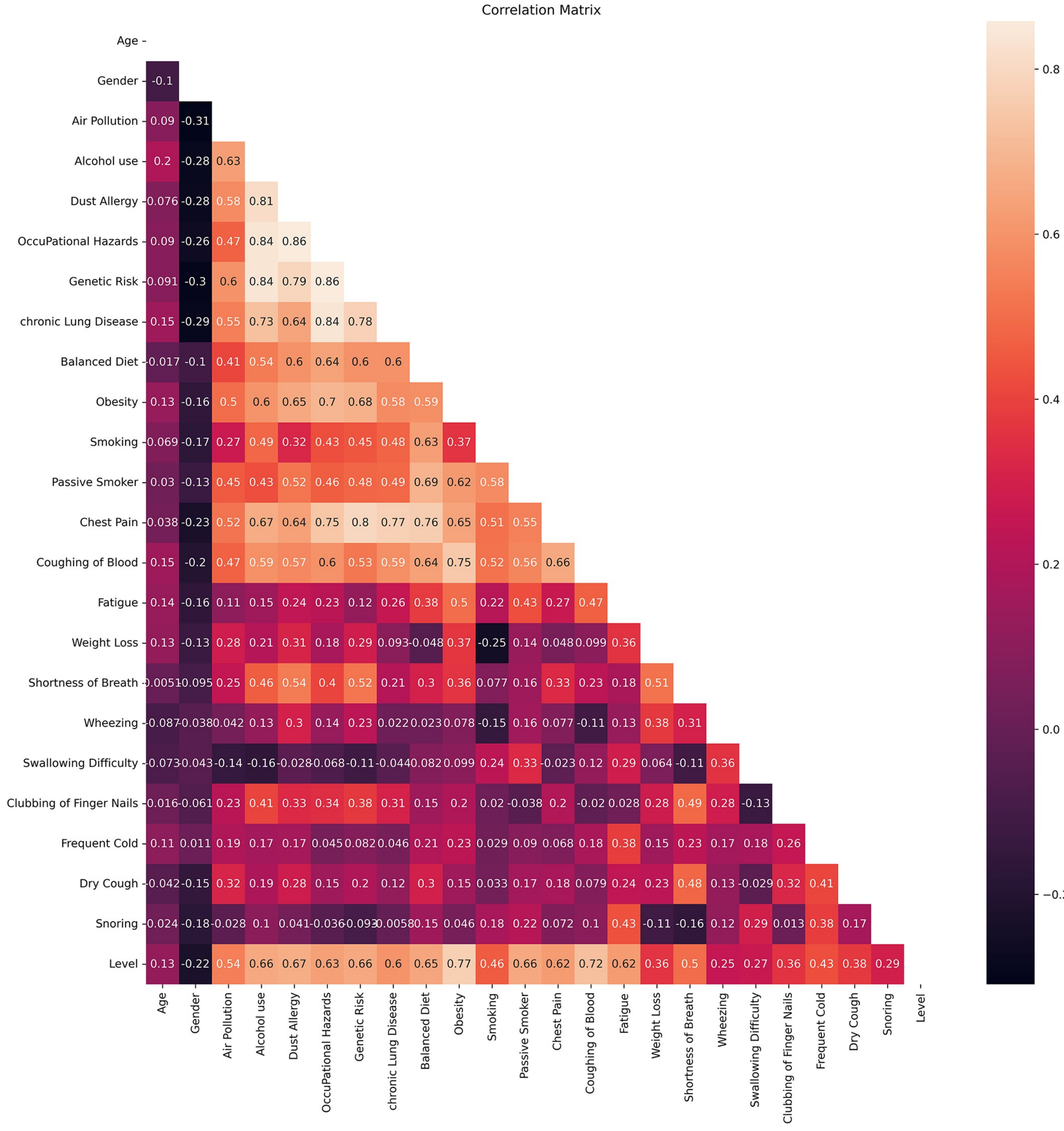

Fig 3. Correlation matrix heatmap.

https://doi.org/10.1371/journal.pone.0305035.g003

Parameter tuning has been applied to select the best parameters among given dictionaries using the Grid Search algorithm with cv = 5 and n_jobs = 5. The Fbeta scorer was selected with beta = 2, and the micro average was considered to create the scorer. For SVM, four parameters were given with suitable ranges as C = [0.1,1,10]; Kernel = [rbf, poly, sigmoid,

Table 2. Dataset distribution between train and test sets.

	Low	Medium	High	Total
Training	219	235	246	700
Testing	84	97	119	300
Total	303	332	365	1000

https://doi.org/10.1371/journal.pone.0305035.t002

linear]; class weight = [balanced, None] and decision function shape = [ovo, ovr]. Similarly, for KNN given parameters and values are, n neighbors = [3,5,7], weights = [uniform, distance], algorithm = [auto, ball_tree, kd_tree, brute], leaf size = [10, 20, 30, 40], p = [1, 2].For DT, given parameters are, criterion = [gini, entropy], splitter = [best, random], max depth = [10, 20, 30, None], min samples split = [2, 5, 10], min samples leaf = [1, 2, 4], max features = [auto, sqrt, log2]. Finally, for RF classifier given parameters are, n estimators = [10, 20, 30], max features = [auto, sqrt, log2], max depth = [10, 20, 30, None], criterion = [gini, entropy] and class weight = [balanced, balanced subsample, None]. Though parameter tuning has also been tested on DT and RF, as they are already achieving 100% accuracy, after tuning, parameters are just the first value of the dictionary. Improved parameters after grid search algorithm that are applied: {'C': 0.1, 'class_weight': 'balanced', 'decision_function_shape': 'ovo', 'kernel': 'linear'} for SVM and {'algorithm': 'auto', 'leaf_size': 10, 'n_neighbors': 3, 'p': 1, 'weights': 'uniform'} for KNN. All four models have been trained again with selected parameters, and this time, SVM, KNN, and DT showed improved K-fold test accuracy.

Table 3. Feature importance values extracted using RF.

Feature name	Importance Value
1. Coughing of Blood	0.123877
2. Passive Smoker	0.114201
3. Wheezing	0.081368
4. Alcohol use	0.069177
5. Fatigue	0.063178
6. Obesity	0.061386
7. Smoking	0.055678
8. Dust Allergy	0.052402
9. Shortness of Breath	0.051129
10. Swallowing Difficulty	0.042071
11. Occupational Hazards	0.039309
12. Balanced Diet	0.034404
13. Chest Pain	0.034277
14. Air Pollution	0.033687
15. Frequent Cold	0.026834
16. Clubbing of Fingernails	0.025768
17. Snoring	0.022114
18. Weight Loss	0.022050
19. Dry Cough	0.019524
20. Chronic Lung Disease	0.013363
21. Genetic Risk	0.011912
22. Age	0.002291
23. Gender	0.000000

https://doi.org/10.1371/journal.pone.0305035.t003

Model explainability

Machine Learning model explainability refers to the understanding and interpreting how a model arrives at its predictions, in this case, risk levels. It is about demystifying the black-box nature of complex scenarios and making their internal functionality more transparent and understandable to humans. Understanding why a model makes a specific prediction or classification can help build trust in its reliability and fairness and provide valuable insights for improving the model's performance or addressing biases.

There are various techniques for enhancing model explainability, ranging from simple methods like feature importance analysis to more sophisticated approaches such as generating human readable explanations for individual predictions using popular algorithms like LIME or Shapley Additive Explanations (SHAP).

Support vector machine (SVM). A decision boundary has been plotted and analyzed to explain the outcome of the SVM model and shown in Fig 4. However, a decision boundary is a 2D plot, where the dataset has multiple features; hence, a principal component analysis (PCA) was done to reduce the dimensionality of the data. After doing 2D PCA, minimum and maximum values are extracted to create a mesh grid. SVM has been trained to get the Z-axis values using that grid data. A decision boundary has been drawn using the mesh grid and Z-axis values, and a scatter plot is used to plot the training and testing data points.

K-nearest neighbors (KNN). The LIME method has been used to explain the KNN model. LIME begins by generating a dataset of perturbed instances around the instance of interest. For presenting a prediction made by a KNN model, LIME would generate new data points by perturbing the features of the instance being explained. The KNN model is then used to predict the output for these perturbed instances. Since KNN is a lazy learner, it directly uses the nearest neighbors from the training data to make predictions without explicitly building a model. Next, LIME fits an interpretable model to the generated dataset using the perturbed

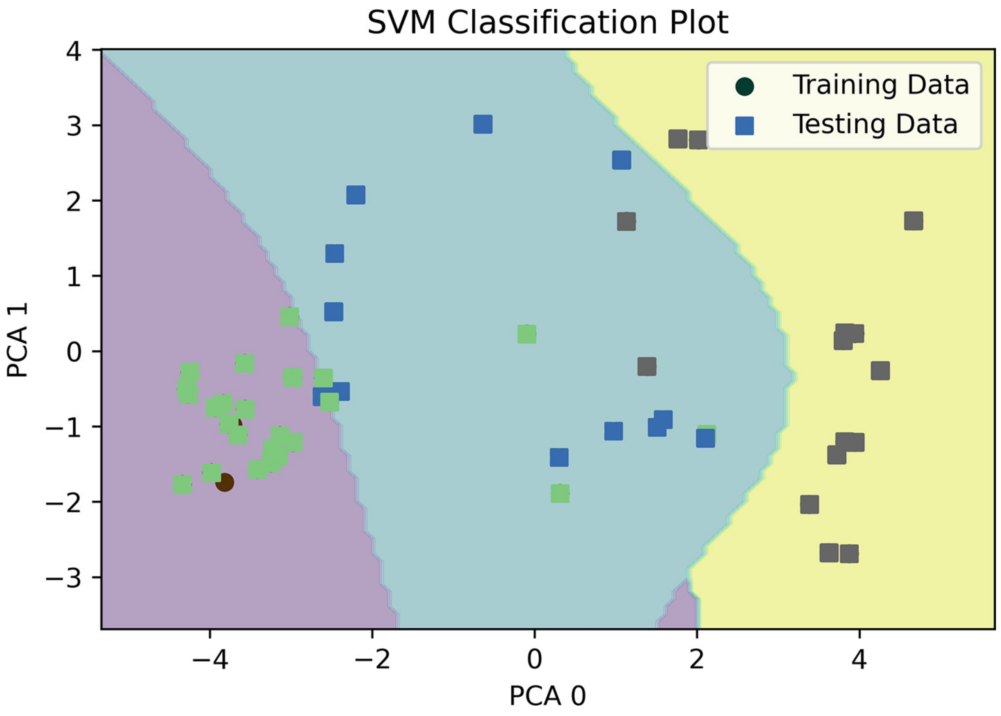

Fig 4. Decision boundary plotting for SVM.

https://doi.org/10.1371/journal.pone.0305035.g004

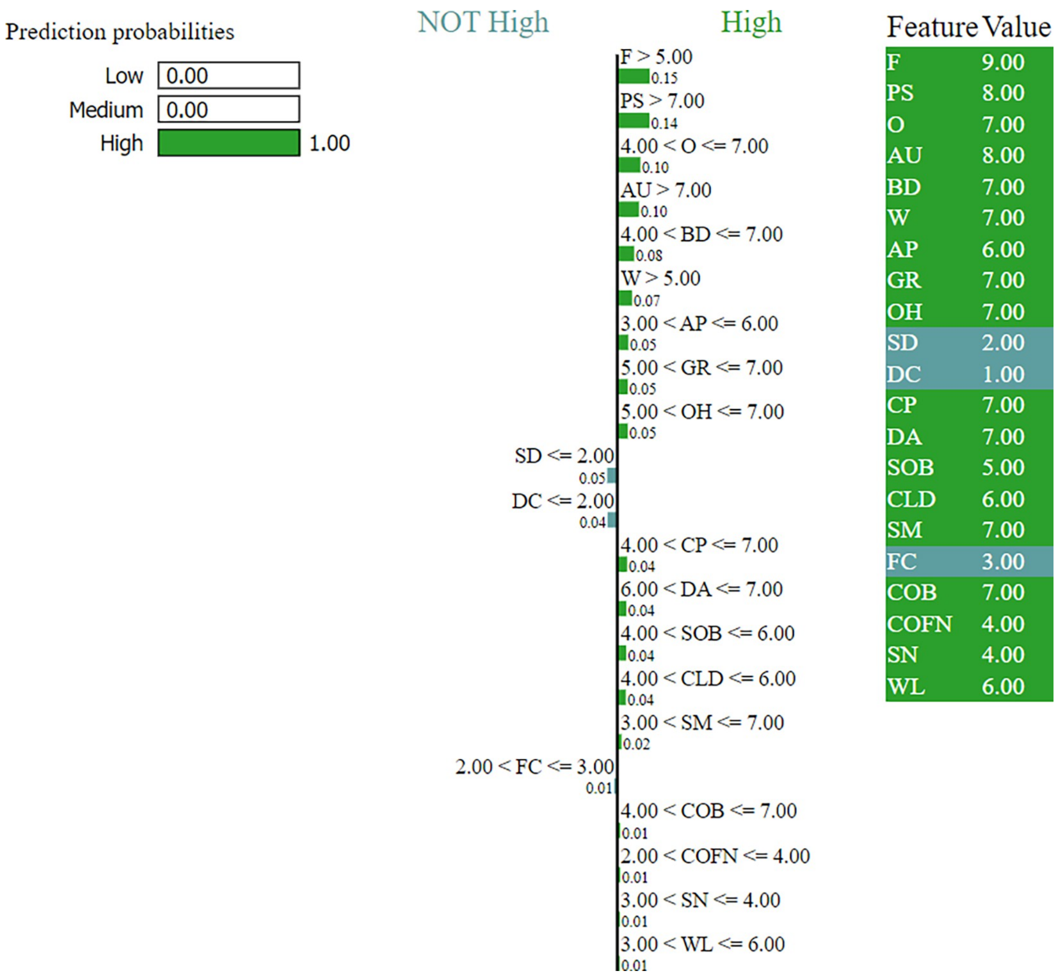

Fig 5. LIME explanation of a "High" risk class.

https://doi.org/10.1371/journal.pone.0305035.g005

instances and the corresponding predictions obtained from the KNN model. The coefficients or feature importances of the interpretable model trained in the previous step are used to determine the contribution of each feature to the KNN model's prediction for the instance of interest. This helps in understanding which features are most influential in the decision made by the KNN model. Finally, LIME provides an explanation for the prediction by highlighting the most essential features and their contributions. This explanation helps us understand why the KNN model made a particular prediction for the instance under consideration.

One sample is shown from a targeted three-level class for better understanding. An explanation of a "High" risk prediction that KNN does is shown in Fig 5, where influential parameters and their value ranges are demonstrated with feature value. In these graphs, full feature names have been shortened due to the width limitation of the plot; the details abbreviations are: 'AP' = > 'Air Pollution', 'AU' = > 'Alcohol use', 'DA' = > 'Dust Allergy', 'OH' = > 'Occupational Hazards', 'GR' = > 'Genetic Risk', 'CLD' = > 'chronic Lung Disease', 'BD' = > 'Balanced Diet', 'O' = > 'Obesity', 'SM' = > 'Smoking', 'PS' = > 'Passive Smoker', 'CP' = > 'Chest Pain', 'COB' = > 'Coughing of Blood', 'F' = > 'Fatigue', 'WL' = > 'Weight Loss', 'SB' = > 'Shortness of Breath', 'W' = > 'Wheezing', 'SD' = > 'Swallowing Difficulty', 'COFN' = > 'Clubbing of Finger Nails', 'FC' = > 'Frequent Cold', 'DC' = > 'Dry Cough' and 'SN' = > 'Snoring'.

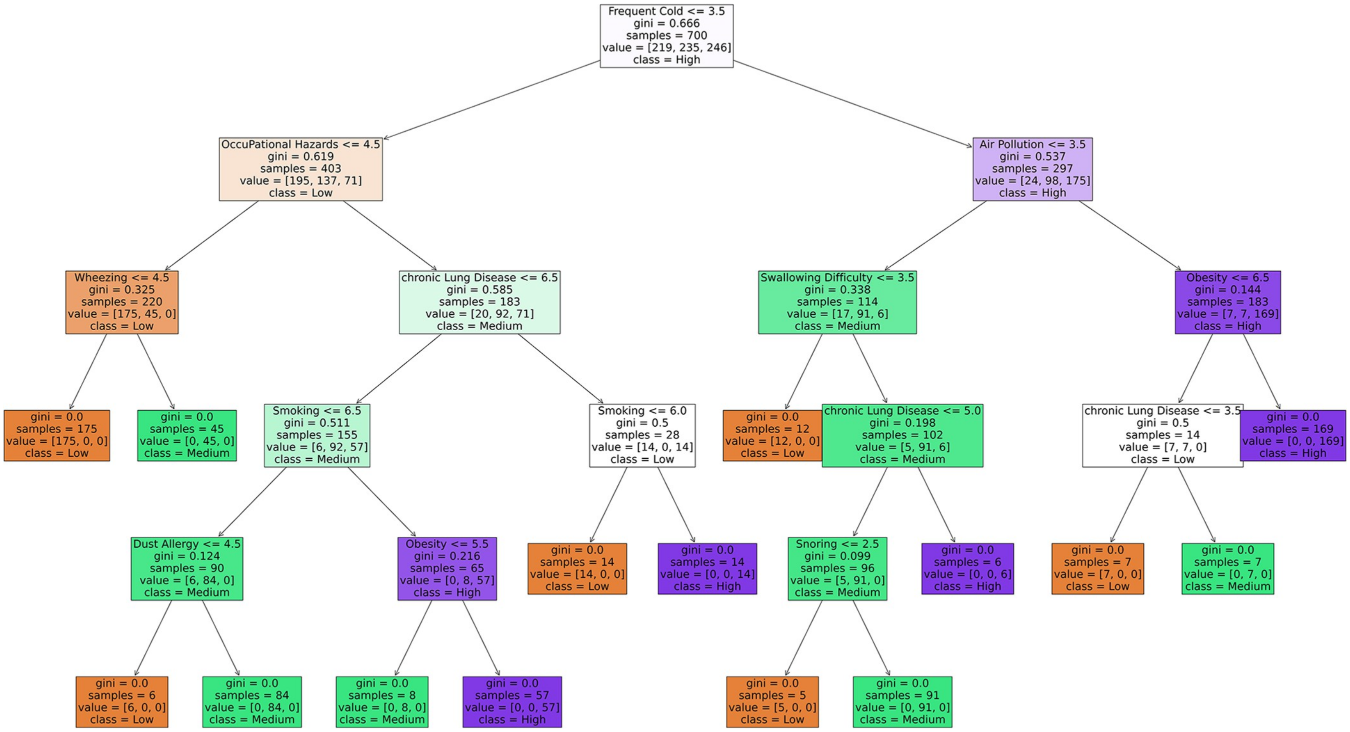

Fig 6. Decision tree explanation.

https://doi.org/10.1371/journal.pone.0305035.g006

Decision tree. A decision tree is a popular supervised learning algorithm for classification and regression tasks. It works by recursively partitioning the input space into smaller regions and assigning a label or value to each area. This process creates a tree-like structure where each internal node represents a decision based on a feature, each branch represents a possible outcome of that decision, and each leaf node represents the final prediction or value. The topmost node in the tree is the root node, representing the entire input space. Nodes that represent decisions based on features are called internal nodes. Each internal node splits the data into two or more subsets based on a feature value. The edges connecting nodes are called branches, representing the possible outcomes of decisions. Nodes at the end of the branches that do not split further are terminal nodes. They represent the final predictions or values. At each internal node, the decision tree algorithm selects the best feature and the corresponding threshold to split the data into subsets. The goal is to maximize the homogeneity or purity of the subsets regarding the target variable. After selecting the splitting criteria, the algorithm recursively applies the splitting process to each subgroup, creating a binary tree structure. The recursive partitioning process continues until a stopping criterion is met. Standard stopping criteria include reaching a maximum tree depth, achieving a minimum number of samples per leaf node, or no further improvement in homogeneity. Once the tree is constructed, it can be used to make predictions for new instances by traversing the tree from the root node to a leaf node based on the values of the input features. In our case, Frequent Cold is the root node that links to Air Pollution and Obesity for direct high-risk prediction. A complete tree explanation has been shown in Fig 6.

Random forest. Plotting a decision tree from a random forest ensemble can provide insights into how the individual decision trees within the random forest make predictions collectively. While each decision tree in a random forest is trained independently, understanding

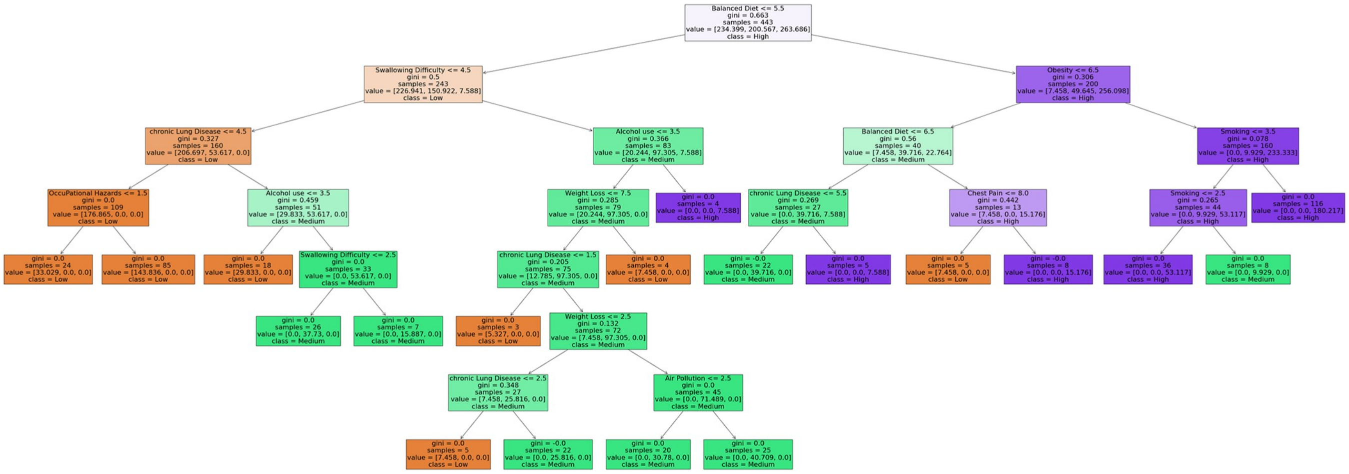

Fig 7. One tree is represented by the random forest model.

https://doi.org/10.1371/journal.pone.0305035.g007

the structure of a single decision tree can help in understanding the overall behavior of the random forest algorithm. However, it's important to note that plotting a single decision tree from a random forest does not fully represent the complexity and diversity of the entire ensemble. Each decision tree in a random forest is trained on a bootstrapped subset of the original dataset and may use a random subset of features at each split. Plotting an individual decision tree can help understand the specific features and decision criteria used by that tree to make predictions. By analyzing the splits and decisions made by the particular tree, one can infer the importance of different features in making predictions. Features that appear higher in the tree and are used for multiple splits are likely more important in decision-making. While a single decision tree provides insights into the decision-making process, the strength of random forests lies in aggregating predictions from multiple trees. Plotting multiple decision trees from a random forest and analyzing their commonalities and differences can help understand how the ensemble combines diverse predictions to improve overall accuracy and generalization. Understanding the decision-making process of individual trees within a random forest can enhance the interpretability of the model, providing insights into why specific predictions are made and how different features contribute to those predictions. The first decision tree from the random forest model is shown in Fig 7.

Result analysis

Initially, all four models are trained with default parameters, having five k-fold cross-validation. SVM has achieved a cross-validation accuracy of 95% (+/- 0.02), total accuracy of 96.33%, KNN achieved a cross-validation accuracy of 92% (+/- 0.07), and total accuracy of 99.66%. On the other hand, the decision tree achieved 99% (+/- 0.03) cross-validation accuracy, and the random forest achieved 100% accuracy in cross-validation and total accuracy. Detailed results, including precision, recall, and f1-score are shown in Table 4 for all four models. The confusion matrix of the test result is shown in Fig 8.

Though the classification accuracy with tree algorithms is highest at 100%, SVM seems to miss some values. For which tuned parameters are seen as an improvement in accuracy, achieving almost 100% test accuracy even with five k-folding tests. Similar improvements have been seen for KNN, which has come to 99% test accuracy from the previous 92%, improving almost 7%. The decision tree also improved on the 1st K-fold test, increasing to 100% from

Table 4. Detailed results of four ML models on the overall test dataset.

Model	Classes	Precision	Recall	F1-score	Support	Accuracy
SVM	0	1.00	0.90	0.95	84	0.96
	1	0.92	0.97	0.94	97	
	2	0.98	1.00	0.99	119	
KNN	0	1.00	0.99	0.99	84	0.99
	1	0.99	1.00	0.99	97	
	2	1.00	1.00	1.00	119	
DT	0	1.00	1.00	1.00	84	1.00
	1	1.00	1.00	1.00	97	
	2	1.00	1.00	1.00	119	
RF	0	1.00	1.00	1.00	84	1.00
	1	1.00	1.00	1.00	97	
	2	1.00	1.00	1.00	119	

https://doi.org/10.1371/journal.pone.0305035.t004

96%, but decreased 1% on the 3rd K-fold test, but overall remains at 99% test accuracy. The random forest model remains unchanged on all K-folding. A detailed comparison of results is shown in Table 5. The learning curve has also been analyzed before and after parameter tuning. Before parameter tuning, SVM shows a slow learning curve in training and cross-validation. Similar but lower validation scores can also be seen with KNN. Comparatively, DT and RF show better training and cross-validation scores from the beginning. Learning curves for four models before parameter tuning have been shown in Fig 9.

After parameter tuning, much better learning curves are seen for SVM and KNN. Previously, reaching the peak accuracy took SVM around 400 training sets, but now it is achieved within 300 training sets. Similarly, KNN's previous peak performance was at 500 training sets, but now it is achieved within 400 training sets. Little performance disruption is seen for DT, but it can ultimately maintain its peak accuracy. No noticeable changes were seen for RF, as it has a stable learning curve and accuracy throughout the K-folding and fixed parameter tests. Details of learning curves after parameter tuning for four models are shown in Fig 10.

Discussion

Machine learning has always been one of the most popular techniques for analyzing numeric data. Specifically, it has been widely used in medical science to help healthcare professionals with prognosis, diagnosis, different factor analysis, and so on. In this paper, four ML models are used to predict lung cancer risk levels, as well as analyze the explainability of these models' outcomes. Previous studies that worked with similar datasets used some standard models but achieved lower performance and provided no logical explanation [22, 26]. Ahmad A. et al. even collected expert professional comments to compare with the ML predicted result, which shows the superiority of ML prediction. Even with CT scans, the CNN method shows better accuracy than senior doctors in the case of risk assessment [33]. It refers to the importance of explainability in answering why these models can perform better than professionals.

This paper worked with a lung cancer risk assessment dataset, which has been trained with four machine learning models, achieving higher accuracy than previous work [26]. Hyperparameter tuning has been done using the Grid Search algorithm to get the best parameters among given ranges for all four models. After training with those parameters, improvements were seen in the SVM, KNN, and DT models. Random Forest has remained the top scorer since the beginning due to its capability of generating multiple trees, each having split features based on feature weights. SVM, KNN, and DT are some of the most popular models for training

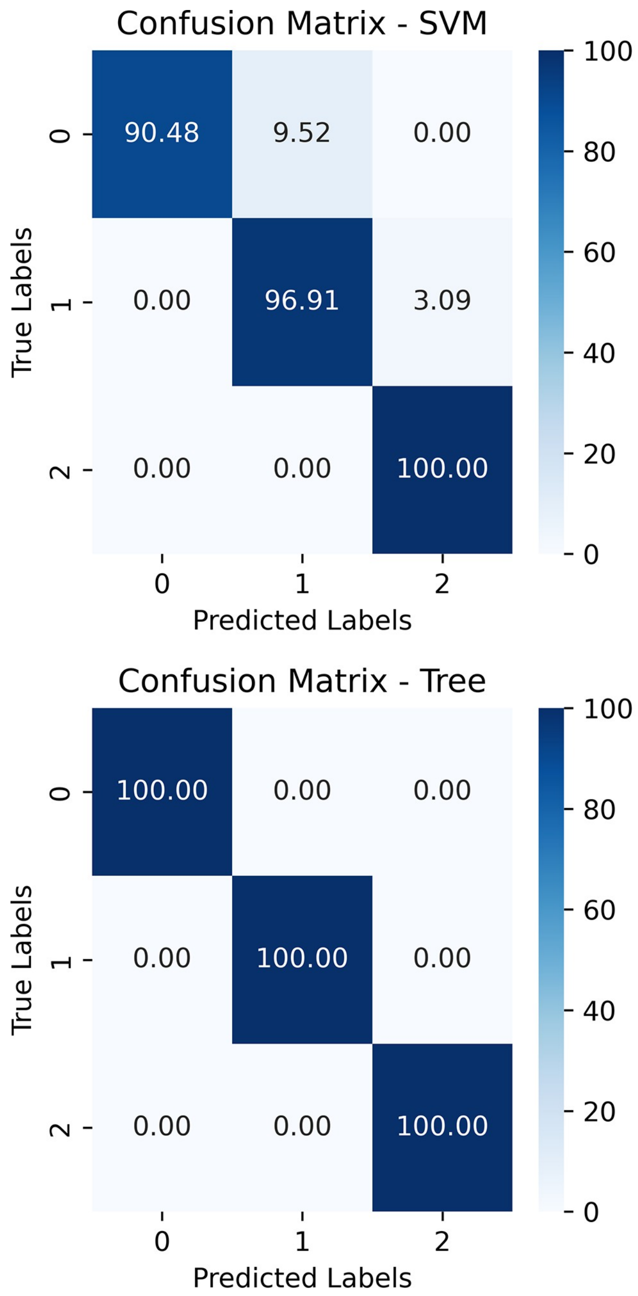

Fig 8. A confusion matrix shows the classification result (in %) of test data using four ML models.

https://doi.org/10.1371/journal.pone.0305035.g008

numeric datasets, and some other Kaggle datasets were seen to be trained by using these models. One recent work achieved accuracy around 95.4% for SVM, 93.7% for DT, and 95.2% for KNN [21], another work [22] achieved 99.2% with SVM and 90% with DT on the same dataset. In both cases, no hyperparameter tuning has been done, which might be able to increase their accuracy, and no model explanations were mentioned. On a custom-collected dataset, SVM, RF, and KNN were applied to train the data, and they gained 94.7%, 89.5%, and 89.5% model accuracy, respectively. A comparison of these results is shown in Table 6.

Table 5. Comparison of results due to parameter tuning for five k-fold cross-validation.

Model	Tuning	1-fold	2-fold	3-fold	4-fold	5-fold	Accuracy	STD (+/-)
SVM	Before	0.93	0.95	0.96	0.95	0.96	0.95	0.02
	After	1.00	1.00	1.00	0.98	1.00	1.00	0.01
KNN	Before	0.93	0.95	0.92	0.85	0.93	0.92	0.07
	After	1.00	1.00	0.98	1.00	0.98	0.99	0.02
DT	Before	0.96	1.00	1.00	0.98	1.00	0.99	0.03
	After	1.00	1.00	1.00	0.97	1.00	0.99	0.03
RF	Before	1.00	1.00	1.00	0.98	1.00	1.00	0.01
	After	1.00	1.00	1.00	0.98	1.00	1.00	0.01

https://doi.org/10.1371/journal.pone.0305035.t005

This paper also explained the models' performance reasons using different explainability methods. A decision boundary has been drawn for both training and testing data for SVM. From the decision boundary, it can be understood that the data points are well-categorized and differentiable from one class to another. The LIME method has been used to extract the reasoning behind KNN's classification. For example, one "High" risk level category is selected as "High" due to having higher Fatigue, specifically having a value of more than 5, Passive Smoking value of more than 5, Obesity between 4 to 7, Alcohol use value of more than 7, and so on. Similarly, the reasons for other levels can also be determined. For the Decision Tree and Random Forest classifier, a single tree has been shown to explain why the model came to the particular decision of given feature values.

Limitations of the study

While machine learning (ML) models hold promise for lung cancer risk prediction, there are limitations to their efficacy, especially regarding explainability:

Data Dependence: ML models are only as good as the data they are trained on. Biases in the data, such as underrepresentation of certain demographics, can lead to inaccurate predictions for those groups.

Focus on Established Risk Factors: Current models primarily focus on well-established risk factors like smoking history. They might miss subtle or emerging risk factors not yet incorporated into the training data.

False Positives and Negatives: ML models can generate false positives (identifying low-risk individuals as high-risk) and false negatives (missing high-risk individuals). This can lead to unnecessary procedures or missed opportunities for early detection.

Conclusion

Lung cancer remains the deadliest disease, with a high mortality rate throughout the world regardless of economic or social conditions. It is much better to care about early prevention, which could save not only one life but also a whole family. To understand the possibility of lung cancer, its factors are critical to understand and analyze and know early symptoms. In this work, a dataset having 22 such properties have been analyzed. The machine learning models used are very lightweight, easily reproducible, and usable in real life without having much technical knowledge. With parameter tuning, almost 99 to 100% test accuracy has been achieved for all four (SVM, KNN, DT, RF) models, even with 5 K-fold cross-validations. Later, each model's decisions were explained with valid and accessible reasoning through various methods such as decision boundary, LIME, and tree representation. This research has been done considering the low correlation between Age and Gender. This

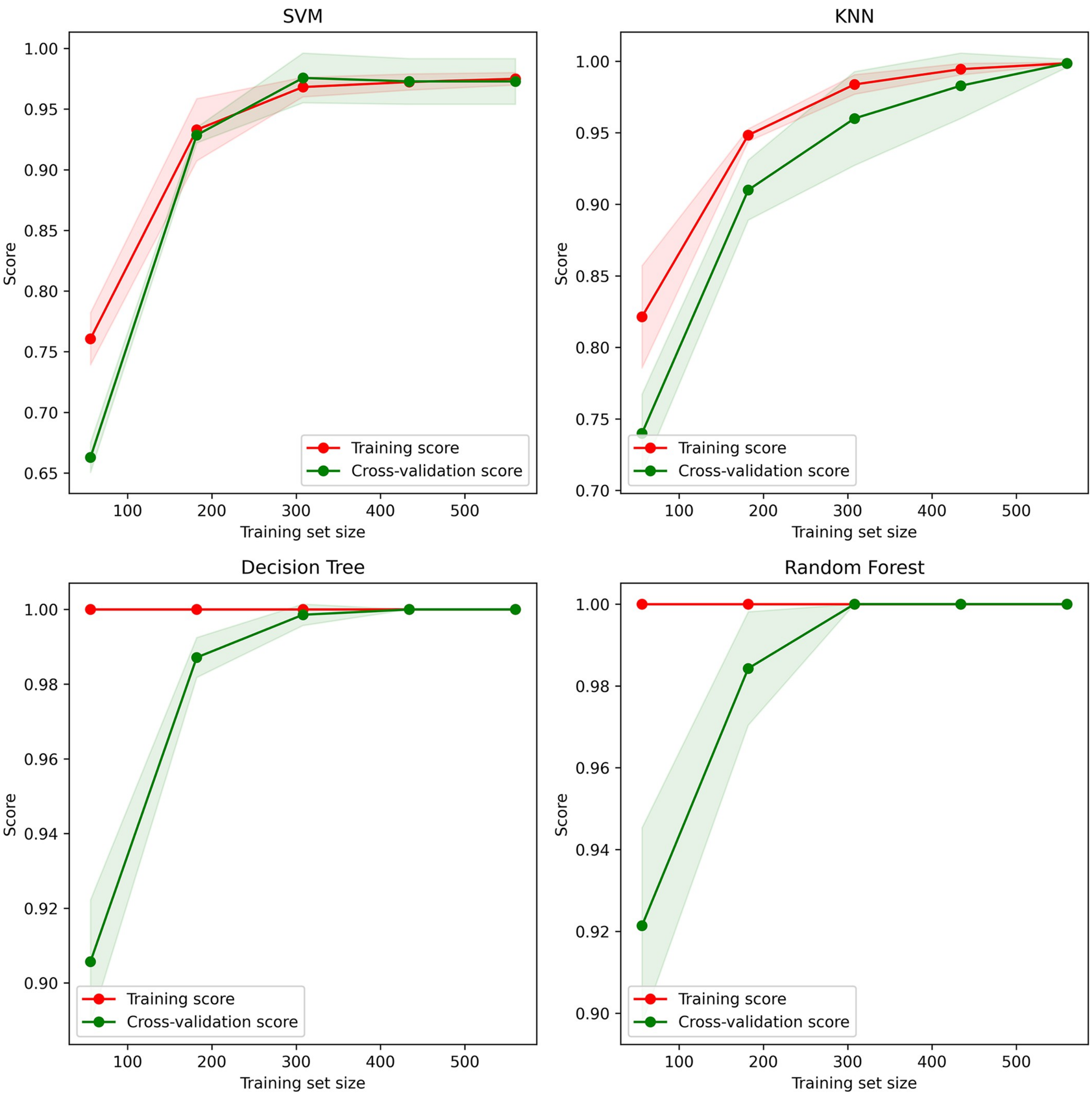

Fig 9. Learning curves for four models before parameter tuning.

https://doi.org/10.1371/journal.pone.0305035.g009

might not be true if any other dataset has a higher correlation. Hence, it can be regarded as a minor limitation. Future work can include multiple neural network-based model architectures to deal with more complex datasets and compare results and explainability with ML models.

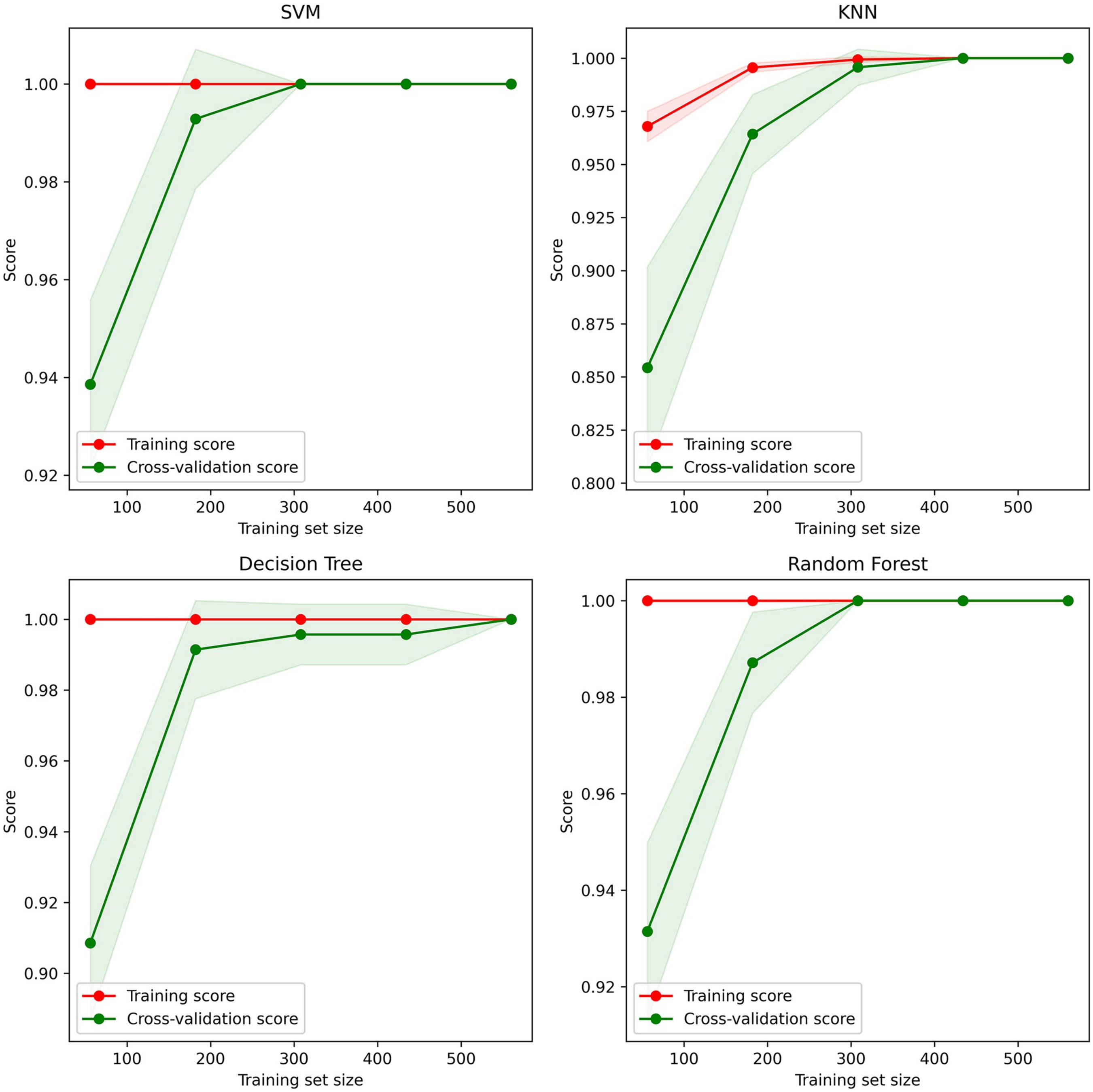

Fig 10. Learning curves after parameter tuning for four models.

https://doi.org/10.1371/journal.pone.0305035.g010

Table 6. Result comparison among literature and this work.

Reference	Dataset	Model	Result in %
[21]	Kaggle—Lung Cancer [43]	SVM	95.4
		ANN	94.6
		NB	95
		DT	93.7
		KNN	95.2
[22]	Data World–Lung Cancer Data [42]	LG	66.7
		DT	90
		NB	87.87
		SVM	99.2
[23]	Kaggle—Lung Cancer DATASET BY STACEYINROBERT [24]	ANN	93
		DT	92.6
[25]	A total of 110 patients and 43 healthy individuals of the Hubei Taihe	KNN	89.5
		SVM	94.7
		RF	89.5
		NN	94.7
	Hospital was included in this study.	NB	100
		AdaBoost	63.2
[26]	Data World–Lung Cancer Data [42]	LCPT	93.33
		Expert Opinion	86.66
This work	Data World–Lung Cancer Data [42]	SVM	100 (+/- 0.01) STD
		KNN	99 (+/- 0.02) STD
		DT	99 (+/- 0.03) STD
		RF	100 (+/- 0.01) STD

https://doi.org/10.1371/journal.pone.0305035.t006

Acknowledgments

The authors express their gratitude to Princess Nourah bint Abdulrahman University Researchers Supporting Project number (PNURSP2024R49), Princess Nourah bint Abdulrahman University, Riyadh, Saudi Arabia.

Author Contributions

Conceptualization: Refat Khan Pathan, Israt Jahan Shorna.

Data curation: Refat Khan Pathan, Israt Jahan Shorna.

Formal analysis: Refat Khan Pathan, Israt Jahan Shorna, Md. Sayem Hossain.

Funding acquisition: Huda I. Almohammed, Zuhal Y. Hamd.

Investigation: Md. Sayem Hossain.

Methodology: Refat Khan Pathan, Md. Sayem Hossain.

Project administration: Mayeen Uddin Khandaker.

Resources: Refat Khan Pathan.

Software: Refat Khan Pathan.

Supervision: Mayeen Uddin Khandaker.

Validation: Refat Khan Pathan, Mayeen Uddin Khandaker.

Visualization: Refat Khan Pathan.

Writing – original draft: Refat Khan Pathan, Israt Jahan Shorna, Md. Sayem Hossain, Mayeen Uddin Khandaker, Huda I. Almohammed, Zuhal Y. Hamd.

Writing – review & editing: Refat Khan Pathan, Israt Jahan Shorna, Md. Sayem Hossain, Mayeen Uddin Khandaker, Huda I. Almohammed, Zuhal Y. Hamd.

References

1. Yount S. et al., "A Brief Symptom Index for Advanced Lung Cancer," *Clin Lung Cancer*, vol. 13, no. 1, pp. 14–23, Jan. 2012, https://doi.org/10.1016/j.cllc.2011.03.033 PMID: 21729652
2. van der Aalst C. M., ten Haaf K., and de Koning H. J., "Lung cancer screening: latest developments and unanswered questions," *Lancet Respir Med*, vol. 4, no. 9, pp. 749–761, Sep. 2016, https://doi.org/10.1016/S2213-2600(16)30200-4 PMID: 27599248
3. ten Haaf K., van der Aalst C. M., de Koning H. J., Kaaks R., and Tammemägi M. C., "Personalising lung cancer screening: An overview of risk-stratification opportunities and challenges," *Int J Cancer*, vol. 149, no. 2, pp. 250–263, Jul. 2021, https://doi.org/10.1002/ijc.33578 PMID: 33783822
4. American Cancer Society, "Key Statistics for Lung Cancer." Jan. 2024. Accessed: Apr. 13, 2024. [Online]. Available: https://www.cancer.org/cancer/types/lung-cancer/about/key-statistics.html
5. American Cancer Society, "Lung Cancer Survival Rates." Jan. 2024. Accessed: Apr. 13, 2024. [Online]. Available: https://www.cancer.org/cancer/types/lung-cancer/detection-diagnosis-staging/survival-rates.html
6. Cancer Research UK, "Lung cancer statistics." Jan. 2024. Accessed: Apr. 13, 2024. [Online]. Available: https://www.cancerresearchuk.org/health-professional/cancer-statistics/statistics-by-cancer-type/lung-cancer
7. Rajadurai P., How S. H., Liam C. K., Sachithanandan A., Soon S. Y., and Tho L. M., "Lung Cancer in Malaysia," *Journal of Thoracic Oncology*, vol. 15, no. 3, pp. 317–323, Mar. 2020, https://doi.org/10.1016/j.jtho.2019.10.021 PMID: 32093853
8. Centers for Disease Control and Prevention, "What Are the Risk Factors for Lung Cancer?" Jul. 2023. Accessed: Apr. 13, 2024. [Online]. Available: https://www.cdc.gov/cancer/lung/basic_info/risk_factors.htm
9. Bach P. B. et al., "Variations in Lung Cancer Risk Among Smokers," *JNCI: Journal of the National Cancer Institute*, vol. 95, no. 6, pp. 470–478, Mar. 2003, https://doi.org/10.1093/jnci/95.6.470 PMID: 12644540
10. Katki H. A., Kovalchik S. A., Berg C. D., Cheung L. C., and Chaturvedi A. K., "Development and Validation of Risk Models to Select Ever-Smokers for CT Lung Cancer Screening," *JAMA*, vol. 315, no. 21, p. 2300, Jun. 2016, https://doi.org/10.1001/jama.2016.6255 PMID: 27179989
11. Cassidy A. et al., "The LLP risk model: an individual risk prediction model for lung cancer," *Br J Cancer*, vol. 98, no. 2, pp. 270–276, Jan. 2008, https://doi.org/10.1038/sj.bjc.6604158 PMID: 18087271
12. Field J. K., Vulkan D., Davies M. P. A., Duffy S. W., and Gabe R., "Liverpool Lung Project lung cancer risk stratification model: calibration and prospective validation," *Thorax*, vol. 76, no. 2, pp. 161–168, Feb. 2021, https://doi.org/10.1136/thoraxjnl-2020-215158 PMID: 33082166
13. Tammemägi M. C. et al., "Selection Criteria for Lung-Cancer Screening," *New England Journal of Medicine*, vol. 368, no. 8, pp. 728–736, Feb. 2013, https://doi.org/10.1056/NEJMoa1211776 PMID: 23425165
14. McRonald F. E. et al., "The UK Lung Screen (UKLS): Demographic Profile of First 88,897 Approaches Provides Recommendations for Population Screening," *Cancer Prevention Research*, vol. 7, no. 3, pp. 362–371, Mar. 2014, https://doi.org/10.1158/1940-6207.CAPR-13-0206 PMID: 24441672
15. Shaheen M. Y., "Applications of Artificial Intelligence (AI) in healthcare: A review," *ScienceOpen Preprints*, 2021, https://doi.org/10.14293/S2199-1006.1.SOR-.PPVRY8K.v1
16. Bi W. L. et al., "Artificial intelligence in cancer imaging: Clinical challenges and applications," *CA Cancer J Clin*, vol. 69, no. 2, pp. 127–157, Mar. 2019, https://doi.org/10.3322/caac.21552 PMID: 30720861
17. Huang S., Yang J., Fong S., and Zhao Q., "Artificial intelligence in cancer diagnosis and prognosis: Opportunities and challenges," *Cancer Lett*, vol. 471, pp. 61–71, Feb. 2020, https://doi.org/10.1016/j.canlet.2019.12.007 PMID: 31830558
18. McKinney S. M. et al., "International evaluation of an AI system for breast cancer screening," *Nature*, vol. 577, no. 7788, pp. 89–94, Jan. 2020, https://doi.org/10.1038/s41586-019-1799-6 PMID: 31894144

19. Pathan R. K. et al., "Breast Cancer Classification by Using Multi-Headed Convolutional Neural Network Modeling," *Healthcare*, vol. 10, no. 12, p. 2367, Nov. 2022, https://doi.org/10.3390/healthcare10122367 PMID: 36553891

20. Ardila D. et al., "End-to-end lung cancer screening with three-dimensional deep learning on low-dose chest computed tomography," *Nat Med*, vol. 25, no. 6, pp. 954–961, Jun. 2019, https://doi.org/10.1038/s41591-019-0447-x PMID: 31110349

21. Dritsas E. and Trigka M., "Lung Cancer Risk Prediction with Machine Learning Models," *Big Data and Cognitive Computing*, vol. 6, no. 4, p. 139, Nov. 2022, https://doi.org/10.3390/bdcc6040139

22. Nair R. P.R., R. A. S, and G V., "A Comparative Study of Lung Cancer Detection using Machine Learning Algorithms," in *2019 IEEE International Conference on Electrical*, *Computer and Communication Technologies (ICECCT)*, IEEE, Feb. 2019, pp. 1–4. https://doi.org/10.1109/ICECCT.2019.8869001

23. Vieira E., Ferreira D., Neto C., Abelha A., and Machado J., "Data Mining Approach to Classify Cases of Lung Cancer," 2021, pp. 511–521. https://doi.org/10.1007/978-3-030-72657-7_49

24. Kannan K.R, "lung cancer DATASET BY STACEYINROBERT." Apr. 2020. Accessed: Apr. 13, 2024. [Online]. Available: https://www.kaggle.com/datasets/imkrkannan/lung-cancer-dataset-by-staceyinrobert

25. Xie Y. et al., "Early lung cancer diagnostic biomarker discovery by machine learning methods," *Transl Oncol*, vol. 14, no. 1, p. 100907, Jan. 2021, https://doi.org/10.1016/j.tranon.2020.100907 PMID: 33217646

26. Ahmad A. S. and Mayya A. M., "A new tool to predict lung cancer based on risk factors," *Heliyon*, vol. 6, no. 2, p. e03402, Feb. 2020, https://doi.org/10.1016/j.heliyon.2020.e03402 PMID: 32140577

27. Thanoon M. A., Zulkifley M. A., Mohd Zainuri M. A. A., and Abdani S. R., "A Review of Deep Learning Techniques for Lung Cancer Screening and Diagnosis Based on CT Images," *Diagnostics*, vol. 13, no. 16, p. 2617, Aug. 2023, https://doi.org/10.3390/diagnostics13162617 PMID: 37627876

28. Lu M. T., Raghu V. K., Mayrhofer T., Aerts H. J. W. L., and Hoffmann U., "Deep Learning Using Chest Radiographs to Identify High-Risk Smokers for Lung Cancer Screening Computed Tomography: Development and Validation of a Prediction Model," *Ann Intern Med*, vol. 173, no. 9, pp. 704–713, Nov. 2020, https://doi.org/10.7326/M20-1868 PMID: 32866413

29. Anthimopoulos M., Christodoulidis S., Ebner L., Christe A., and Mougiakakou S., "Lung Pattern Classification for Interstitial Lung Diseases Using a Deep Convolutional Neural Network," *IEEE Trans Med Imaging*, vol. 35, no. 5, pp. 1207–1216, May 2016, https://doi.org/10.1109/TMI.2016.2535865 PMID: 26955021

30. Crasta L. J., Neema R., and Pais A. R., "A novel Deep Learning architecture for lung cancer detection and diagnosis from Computed Tomography image analysis," *Healthcare Analytics*, vol. 5, p. 100316, Jun. 2024, https://doi.org/10.1016/j.health.2024.100316

31. Huo T. et al., "Deep learning-based algorithm improves radiologists' performance in lung cancer bone metastases detection on computed tomography," *Front Oncol*, vol. 13, Feb. 2023, https://doi.org/10.3389/fonc.2023.1125637 PMID: 36845701

32. Said Y., Alsheikhy A. A., Shawly T., and Lahza H., "Medical Images Segmentation for Lung Cancer Diagnosis Based on Deep Learning Architectures," *Diagnostics*, vol. 13, no. 3, p. 546, Feb. 2023, https://doi.org/10.3390/diagnostics13030546 PMID: 36766655

33. Zhou J. et al., "An ensemble deep learning model for risk stratification of invasive lung adenocarcinoma using thin-slice CT," *NPJ Digit Med*, vol. 6, no. 1, p. 119, Jul. 2023, https://doi.org/10.1038/s41746-023-00866-z PMID: 37407729

34. Zhang Y., Weng Y., and Lund J., "Applications of Explainable Artificial Intelligence in Diagnosis and Surgery," *Diagnostics*, vol. 12, no. 2, p. 237, Jan. 2022, https://doi.org/10.3390/diagnostics12020237 PMID: 35204328

35. Chen D., Zhao H., He J., Pan Q., and Zhao W., "An Causal XAI Diagnostic Model for Breast Cancer Based on Mammography Reports," in *2021 IEEE International Conference on Bioinformatics and Biomedicine (BIBM)*, IEEE, Dec. 2021, pp. 3341–3349. https://doi.org/10.1109/BIBM52615.2021.9669648

36. Munshi R. M., Cascone L., Alturki N., Saidani O., Alshardan A., and Umer M., "A novel approach for breast cancer detection using optimized ensemble learning framework and XAI," *Image Vis Comput*, vol. 142, p. 104910, Feb. 2024, https://doi.org/10.1016/j.imavis.2024.104910

37. Pal M., Mistry S., and De D., "Interpretability Approaches of Explainable AI in Analyzing Features for Lung Cancer Detection," 2023, pp. 277–287. https://doi.org/10.1007/978-981-19-5191-6_23

38. Dwivedi K., Rajpal A., Rajpal S., Kumar V., Agarwal M., and Kumar N., "Enlightening the path to NSCLC biomarkers: Utilizing the power of XAI-guided deep learning," *Comput Methods Programs Biomed*, vol. 243, p. 107864, Jan. 2024, https://doi.org/10.1016/j.cmpb.2023.107864 PMID: 37866126

39. Wani N. A., Kumar R., and Bedi J., "DeepXplainer: An interpretable deep learning based approach for lung cancer detection using explainable artificial intelligence," *Comput Methods Programs Biomed*, vol. 243, p. 107879, Jan. 2024, https://doi.org/10.1016/j.cmpb.2023.107879 PMID: 37897989

40. Kobylińska K., Orłowski T., Adamek M., and Biecek P., "Explainable Machine Learning for Lung Cancer Screening Models," *Applied Sciences*, vol. 12, no. 4, p. 1926, Feb. 2022, https://doi.org/10.3390/app12041926

41. Dwivedi K., Rajpal A., Rajpal S., Agarwal M., Kumar V., and Kumar N., "An explainable AI-driven biomarker discovery framework for Non-Small Cell Lung Cancer classification," *Comput Biol Med*, vol. 153, p. 106544, Feb. 2023, https://doi.org/10.1016/j.compbiomed.2023.106544 PMID: 36652866

42. Prithivraj, "Lung Cancer Data." Apr. 2017. Accessed: Apr. 13, 2024. [Online]. Available: https://data.world/cancerdatahp/lung-cancer-data

43. BHAT MYSAR AHMAD, "Lung Cancer." Apr. 2020. Accessed: Apr. 13, 2024. [Online]. Available: https://www.kaggle.com/datasets/mysarahmadbhat/lung-cancer
